# Order-disorder in room-temperature ionic liquids probed via methyl quantum tunneling

**DOI:** 10.1063/4.0000094

**Published:** 2021-04-05

**Authors:** Eugene Mamontov, Naresh C. Osti, Matthew R. Ryder

**Affiliations:** Neutron Scattering Division, Oak Ridge National Laboratory, Oak Ridge, Tennessee 37831, USA

## Abstract

Room-temperature ionic liquids are promising candidates for applications ranging from electrolytes for energy storage devices to lubricants for food and cellulose processing to compounds for pharmaceutics, biotransformation, and biopreservation. Due to the ion complexity, many room-temperature ionic liquids readily form amorphous phases upon cooling, even at modest rates. Here, we investigate two commonly studied imidazolium-based room-temperature ionic liquids, 1-ethyl-3-methylimidazolium tetrafluoroborate and 1-ethyl-3-methylimidazolium bis(trifluoromethylsulfonyl)imide, as well as their mixtures, to demonstrate how the complex interplay between the crystalline and amorphous phases is affected by the processing conditions, such as thermal history, liquid mixing, and applied pressure. We show that quantum tunneling in the cation methyl groups, measured by high-resolution inelastic neutron scattering, can be used to probe the order-disorder in room-temperature ionic liquids (crystalline vs amorphous state) that develops as a result of variable processing conditions.

## INTRODUCTION

**FIG. 1.**
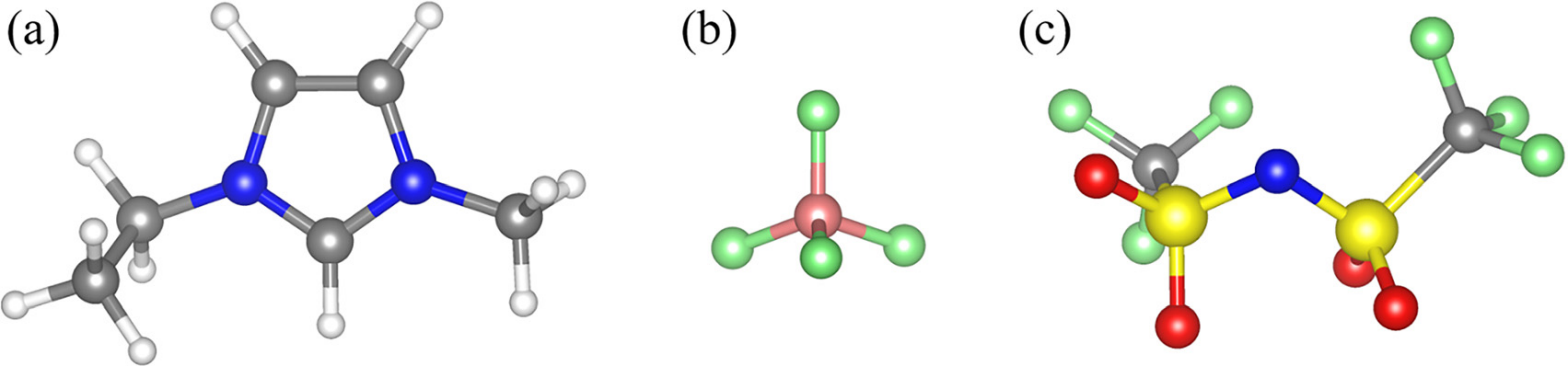
Molecular structures of the (a) Emim cation, and the (b) BF_4_ and (c) TFSI anions as they are oriented in a relaxed triclinic crystal structure similar to that of the bulk materials studied.

Room-temperature ionic liquids (RTILs) are tremendously versatile solvents with a broadly recognized potential for application in numerous fields.^1–10^ More recently, RTILs have attracted much attention for their remarkable affinity for various biomolecules;^11–15^ especially in pharmacology, biomedicine, and bionanotechnology applications, where the behavior of RTILs at low temperatures is of importance. This is because, particularly for biocompatibility and biopreservation, the order or disorder in the structure of the host at low temperature may affect the packing and, thus, the thermal mean-squared displacements of the guest molecules. From both an applied and fundamental research standpoint, RTILs are remarkable in combining ionic conductivity with glass-forming character.^16–18^ Due to the ion complexity, many RTILs readily form amorphous phases upon cooling even at modest rates of 1 K/min or lower, but various factors may play a role in determining whether glass formation or crystallization occurs in a particular sample. Even for crystalline RTIL samples, structural studies may be challenging because of the pronounced polymorphism, with a large number of crystal structures having almost the same energy.^19^ In principle, for structural studies of RTILs with complex organic ions, neutron diffraction from deuterated samples, with the hydrogen (H) atoms replaced by deuterium (D) atoms, would be strongly preferred to x-ray diffraction because of the insensitivity of the latter technique to the hydrogen isotopes. The overwhelming majority of RTILs, however, are not available in the deuterated form, rendering their neutron diffraction studies impossible due to the substantial incoherent neutron scattering background from the H atoms. Hence, the purpose of the current neutron scattering work, for which we chose two extensively studied^20^ imidazolium-based RTILs (Fig. 1), 1-ethyl-3-methylimidazolium tetrafluoroborate (EmimBF_4_) and 1-ethyl-3-methylimidazolium bis(trifluoromethylsulfonyl)imide (EmimTFSI), is to demonstrate how high energy-resolution inelastic neutron scattering (INS) can measure quantum methyl tunneling in RTIL cations to probe the order-disorder (crystalline vs amorphous state) that may develop in RTILs under different conditions. This approach does not require deuterated samples and can be used on any RTIL with methyl-group-containing cations, provided that the quantum tunneling energy of the methyl groups falls into the dynamic range of the high energy-resolution neutron spectrometer utilized for the measurement. Unlike continuous temperature-dependent calorimetric measurements, which may be challenging to interpret when the glass transition is very broad, or difficult to perform if a very high quenching rate is required, INS can directly probe the final state of the sample. In the current work, the continuous temperature-dependent scans of the elastic neutron scattering intensity, which are somewhat analogous to calorimetric measurements, were used to monitor the RTIL samples evolution in response to various applied treatment regimes. The INS results probing the final state of the sample were always in agreement with the elastic intensity scan measurements, thus suggesting that, even when the continuous temperature-dependent monitoring of the sample might be impossible or inconclusive, the order-disorder in the final state of the sample could be probed by INS. Unlike x-ray diffraction, which is sensitive to long-range crystal order, measurements of methyl group tunneling by INS are a probe of the local molecular environment, similar to RTILs measurements by Raman spectroscopy,^21^ nuclear magnetic resonance (NMR),^22^ and EXAFS.^23^ Thus, measurements of methyl group tunneling by INS can potentially be used for probing order-disorder in RTILs without long-range structural order, e.g., in confinement,^24,25^ given the high sensitivity of INS signal to H over heavier elements.

## MATERIALS AND METHODS

The sample preparation of the commercially available EmimBF_4_ and EmimTFSI was the same as described in the earlier neutron scattering study of mixtures of these RTILs for energy storage applications.^20^ Cylindrical annular aluminum sample holders with 29.0 mm outer and 28.9 mm inner diameters, thus providing 0.05 mm thick annular samples, were sealed with indium wire and placed into a top-loading closed-cycle refrigerator (CCR) to control the sample temperature within 0.5 K. In a separate experiment, the EmimBF_4_ sample was pressure-cycled *ex situ* at 5 GPa before the neutron scattering measurements, following the procedure used in an earlier study of pressurized RTILs.^26^ In brief, pressurization to 5 GPa followed by pressure release was carried out in a standard VX3 Paris-Edinburgh cell equipped with single-toroidal cBN anvils and encapsulated TiZr gaskets. The resulting pressure-cycled sample, of limited quantity, was loaded and sealed in a flat plate aluminum holder resulting in a sample thickness of 0.05 mm. Even using the thinnest available sample holder, the amount of the pressure-cycled material was insufficient (77.5 mg) to intercept the entire height of the incident neutron beam of 3 cm, so the resulting scattering signal statistics were lower than the standard (non-pressurized) EmimBF_4_ results.

The high energy-resolution INS measurements were conducted using the time-of-flight backscattering spectrometer, BASIS,^27^ at the Spallation Neutron Source (SNS) facility. The data were Q-averaged for 0.2 Å^−1^ < Q < 1.4 Å^−1^, which provided a full width at half-maximum (FWHM) resolution at the elastic line of ∼3 *μ*eV. The standard instrument setup provided an incident neutron bandwidth covering ±120 *μ*eV energy transfer range. An instrument resolution, which is known to be sample shape-dependent, was measured from annular and flat plate vanadium standards separately for the regular and pressure-cycled sample, respectively.

The standard EmimBF_4_ and EmimTFSI samples were subjected to the following treatment cycles: (1) cooling from the ambient temperature down to ∼5 K at a rate of approximately 1 K/min; (2) warming from 5 K up to ambient temperature at a rate of 1 K/min; (3) removing the sample from the CCR at ambient temperature, cooling the CCR to the baseline temperature, and rapid quenching by inserting the sample into the pre-cooled CCR; (4) warming at a rate of about 1 K/min from 5 to 30 K in an attempt to capture the moment of recrystallization of the glassy sample, then cooling down back to the baseline temperature before the re-crystallized sample could melt. The recrystallization temperatures were chosen based on the data collected in cycle 2. The dynamics measurements were performed at low temperatures after cycles 1, 3, and 4. The pressure-cycled EmimBF_4_ sample was studied in the same manner, except for cycle 3, where the rapid quenching was replaced by regular cooling in the CCR. For the mixtures, only the first two of the four treatment cycles were applied. In most cases, the scattering intensities integrated over ±3 *μ*eV (elastic line defined by the resolution function) were recorded during the cycling. However, on occasion, the sample cycling had to proceed without signal recording because of neutron source downtime to ensure continuous sample processing to measure the dynamics.

## RESULTS AND DISCUSSION

**FIG. 2.**
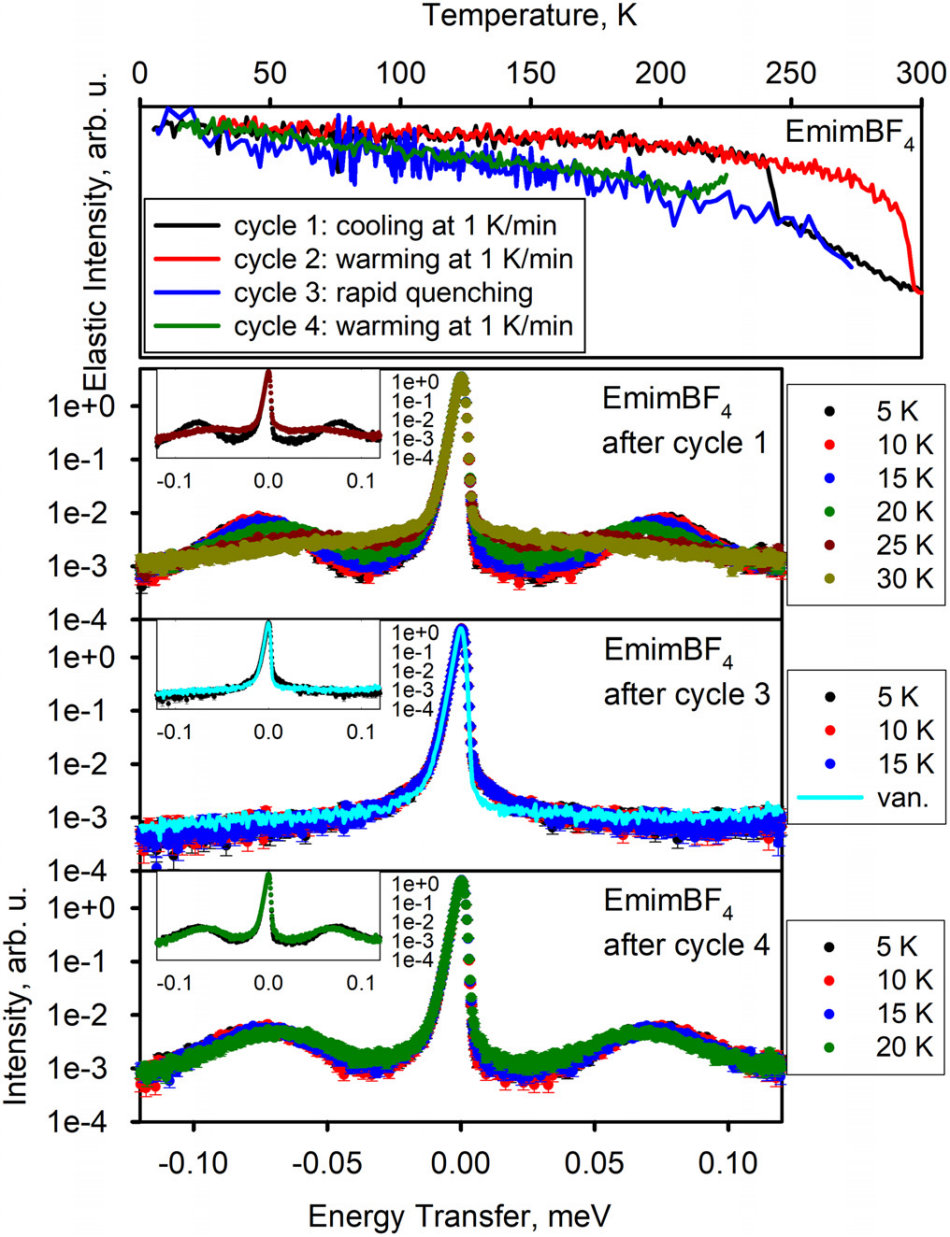
EmimBF_4_. Top panel: elastic neutron scattering intensity as a function of temperature. Bottom panels: dynamics data—neutron scattering intensity as a function of neutron energy transfer measured at selected temperature points. Bottom panels' insets display two selected datasets each; the axes units are the same as in the main panels.

Figure 2 shows the results for EmimBF_4_. On the first cooling cycle 1, there is apparent crystallization just below 245 K, as indicated by a step-like increase in the elastic scattering intensity. The low-temperature dynamics measurements performed after the completion of cycle 1 show peaks centered at around ±75 *μ*eV. These peaks exhibit characteristic properties of quantum methyl rotational tunneling excitations, such as a downward shift and eventual disappearance into the quasi-elastic signal upon increased temperature from ∼30 K. It is evident that the inelastic peaks do not grow in intensity upon increased temperature, as would be the case for vibrational motions obeying Bose–Einstein statistics. We note that the spectra collected at a particular measurement temperature are always represented by the same color for all samples discussed below (e.g., black for 5 K, red for 10 K, and so on).

The quantum methyl tunneling peaks are similar to those reported at ±27 *μ*eV for the RTIL [DMIm][TFSI] in the presence of bis(trifluoromethane)sulfonimide lithium salt, which also did not become amorphous and instead crystallized upon cooling.^19^ The Emim cations in EmimBF_4_ and EmimTFSI have a methyl and an ethyl group. The temperature dependence of the elastic scattering intensity collected upon warming (cycle 2) follows the same pattern observed when cooling (cycle 1), except that the melting step is not detected until ∼290 K. The data collected during the rapid quenching (cycle 3) exhibit more noise because of the shorter collection time per data point in the course of the uncontrolled quenching. The absence of a crystallization-related step-like elastic intensity increase is evident, suggesting glass formation instead of crystallization. Indeed, there are no tunneling peaks in the low-temperature dynamics measurements performed after cycle 3. Comparison with the resolution spectrum collected from a vanadium standard shows that instead of tunneling peaks, the 5, 10, and 15 K spectra now exhibit a signal that looks like quasi-elastic scattering but is practically temperature independent. This behavior is indicative of tunneling in the disordered glassy state.^28–31^ Finally, the elastic scattering intensity was collected on the controlled warming (cycle 4) from a low temperature of 30 K up to ∼210 K from the quenched sample measured in cycle 3. Cycle 4 was stopped when the intensity started to increase between 210 and 220 K. The increase in intensity indicates possible recrystallization of the glassy sample.^32^ Indeed, dynamics measurements performed after cooling upon the completion of cycle 4 show restoration of the tunneling peaks as measured after cycle 1, thus indicating recrystallization of the glassy quenched sample obtained after cycle 3 once it was above 210 K in cycle 4. Because the peaks are at the same position as after cycle 1, it is likely that the crystal structure attained after cycle 4 is the same as after cycle 1.

**FIG. 3.**
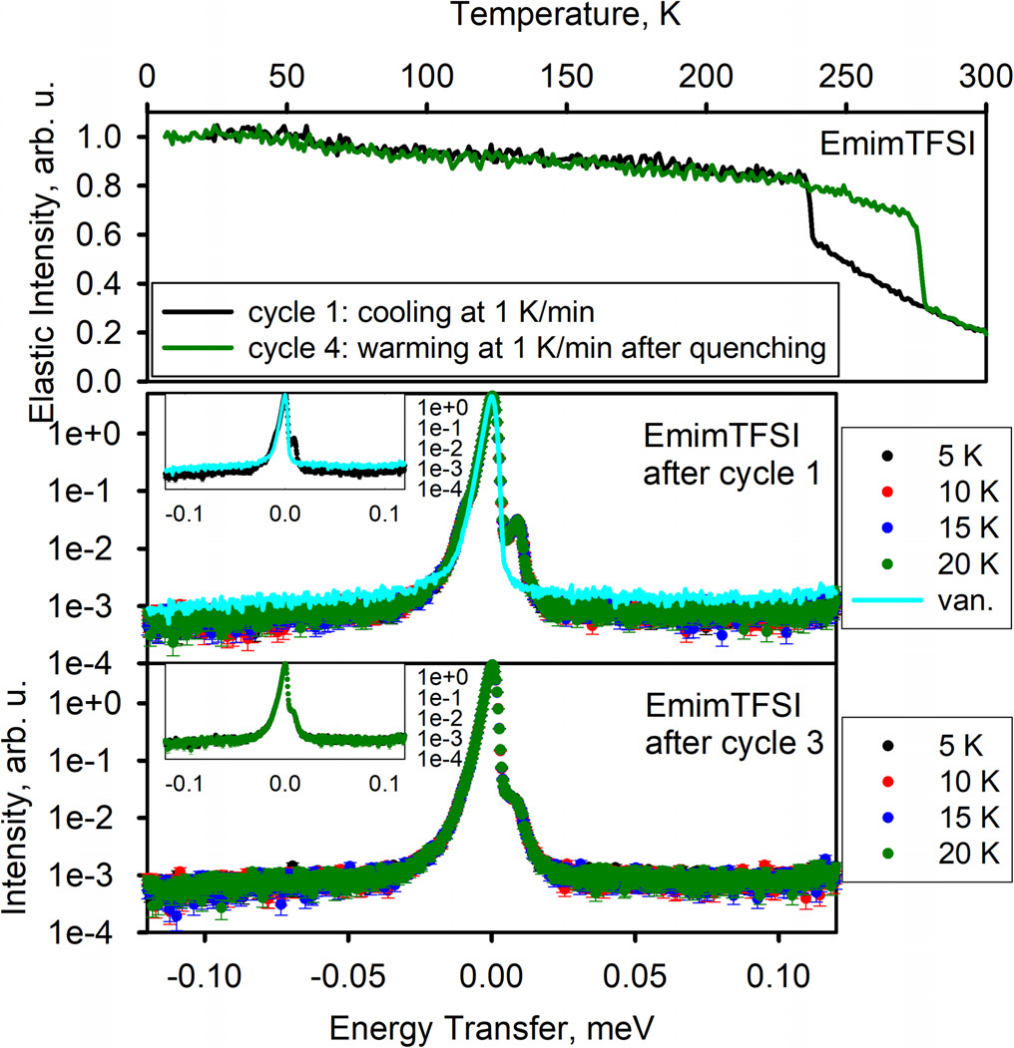
EmimTFSI. Top panel: elastic neutron scattering intensity as a function of temperature. Bottom panels: dynamics data—neutron scattering intensity as a function of neutron energy transfer measured at selected temperature points. Bottom panels' insets display two selected datasets each; the axes units are the same as in the main panels.

Figure 3 shows the results for EmimTFSI. On the first cooling cycle 1, there is apparent crystallization just below 240 K, as indicated by a step-like increase in the elastic scattering intensity. The low-temperature dynamics measurements performed after the completion of cycle 1 show peaks around ±10 *μ*eV (the signal at −10 *μ*eV is obscured by the asymmetric resolution function defining the elastic peak). Due to the proximity of the signals to the elastic line, the temperature behavior is challenging to discern. We assign these peaks to methyl rotational tunneling in crystallized EmimTFSI, similar to those observed in EmimBF_4_, but with a higher potential energy barrier, hence the lower peak position, compared to EmimBF_4_. Although cycle 2 (warming) and cycle 3 (quenching) were carried out when the neutron source was down, the dynamics data collected after cycle 3 in EmimTFSI show a significant difference compared to the dynamics data collected after the completion of cycle 3 in EmimBF_4_.

The EmimTFSI dynamics data still exhibit the methyl quantum tunneling peaks, whereas the EmimBF_4_ data showed a quasi-elastic-looking signal due to the methyl quantum tunneling in the disordered state, as discussed earlier. This suggests that unlike in EmimBF_4_, the quenching attempt failed to produce a glassy EmimTFSI sample, which crystallized instead, giving rise to the well-defined tunneling peaks. The peaks in EmimTFSI after the completion of cycle 3 are somewhat challenging to define due to the proximity to the elastic line; however, the elastic scattering intensities collected on the controlled warming (cycle 4) strongly corroborate the conclusion that EmimTFSI crystallized and did not become glassy upon the attempted quenching (cycle 3). This is because, in variance with EmimBF_4_, the elastic scattering intensities from EmimTFSI collected during cycle 4 overlap well with those collected during cycle 1, and unlike for EmimBF_4_, do not exhibit a sudden increase upon warming, which was indicative of recrystallization in EmimBF_4_. Essentially, the elastic scattering intensities from EmimTFSI collected during cycle 4 were qualitatively similar to those collected from EmimBF_4_ during cycle 2, indicating warming from the crystalline, not glassy, state and eventual melting with no prior recrystallization. Therefore, unlike in EmimBF_4_, the quenching attempt failed to produce a glassy EmimTFSI sample. Instead, the EmimTFSI sample crystallized.

**FIG. 4.**
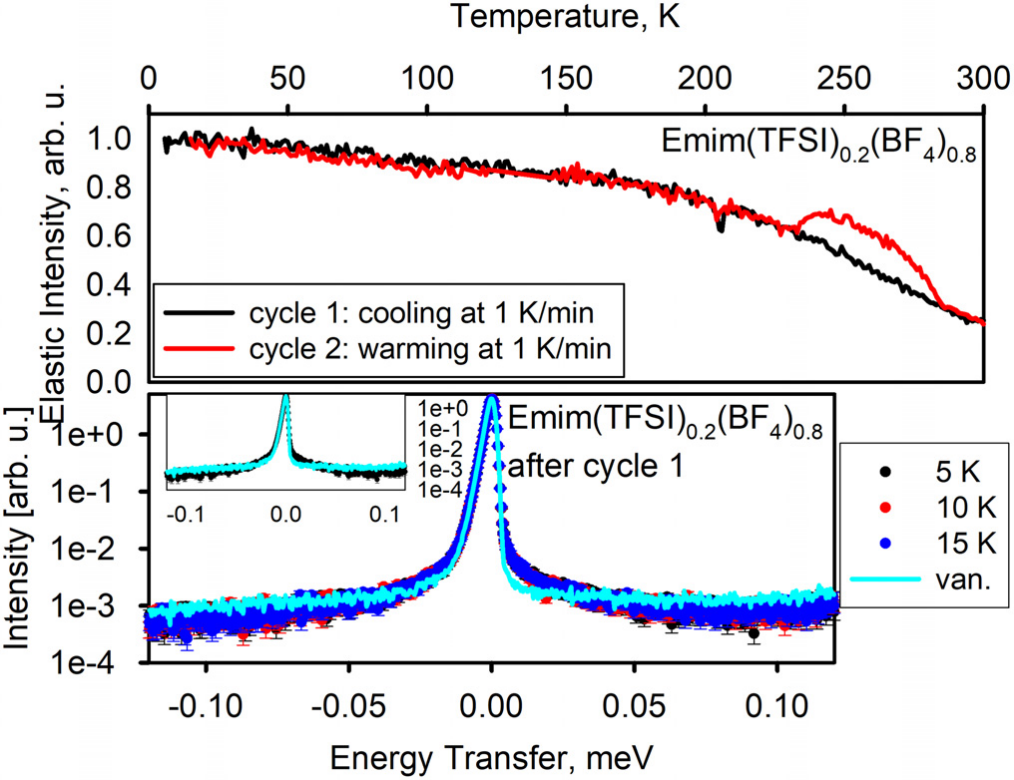
Emim(TFSI)_0.2_(BF_4_)_0.8_. Top panel: elastic neutron scattering intensity as a function of temperature. Bottom panel: dynamics data—neutron scattering intensity as a function of neutron energy transfer measured at selected temperature points. Bottom panel inset displays two selected datasets; the axes units are the same as in the main panel.

Figure 4 shows the results for Emim(TFSI)_0.2_(BF_4_)_0.8_, for which only cycle 1 (cooling) and cycle 2 (warming) were carried out. Unlike with EmimBF_4_ and EmimTFSI, there was no crystallization step in the elastic intensity data collected from Emim(TFSI)_0.2_(BF_4_)_0.8_ upon cooling. The dynamics measurement performed after the completion of cycle 1 also shows that similar to EmimBF_4_ after quenching (cycle 3), Emim(TFSI)_0.2_(BF_4_)_0.8_ after cooling (cycle 1) exhibits a signal that looks like quasi-elastic scattering but is practically temperature independent, as evident from a comparison with the resolution spectrum collected from a vanadium standard. This is indicative of tunneling in the disordered glassy state.^28–31^ Finally, the elastic scattering intensity data collected upon controlled warming (cycle 2) matched that measured for cycle 1 up to ∼230 K, then exhibited the upward trend between 230 and 250 K, indicating recrystallization of the glassy sample,^32^ similar to EmimBF_4_ as described earlier.

**FIG. 5.**
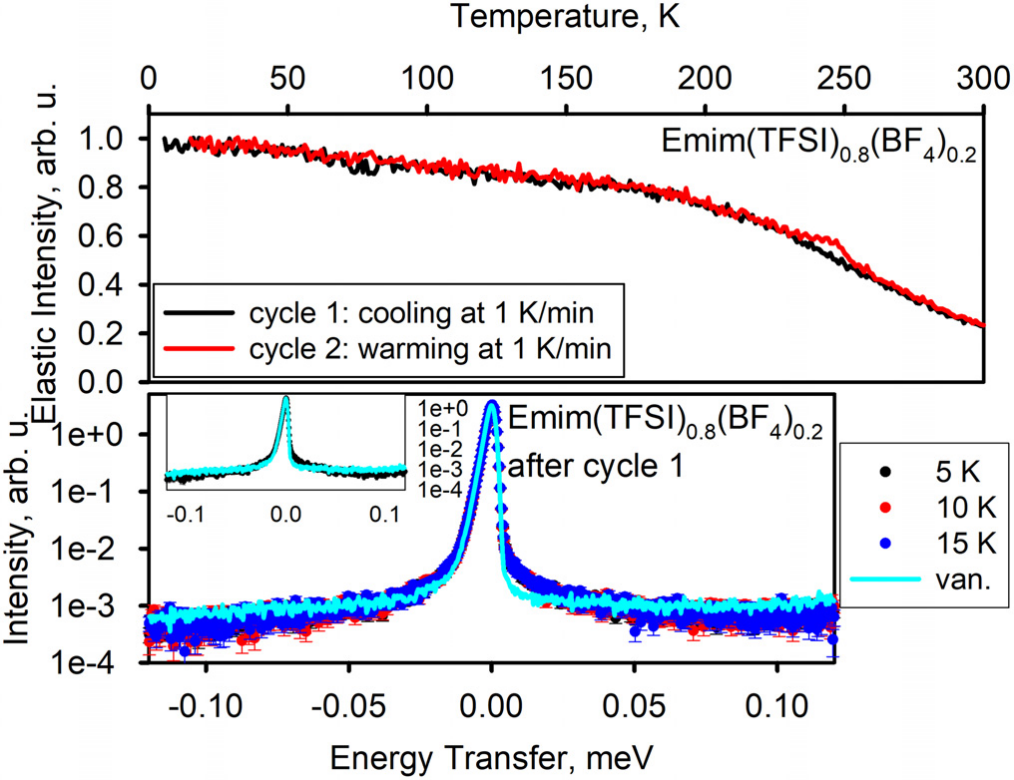
Emim(TFSI)_0.8_(BF_4_)_0.2_. Top panel: elastic neutron scattering intensity as a function of temperature. Bottom panel: dynamics data—neutron scattering intensity as a function of neutron energy transfer measured at selected temperature points. Bottom panel inset displays two selected datasets; the axes units are the same as in the main panel.

Figure 5 shows the results for Emim(TFSI)_0.8_(BF_4_)_0.2_, for which only cycle 1 (cooling) and cycle 2 (warming) were carried out. The results resemble those observed for Emim(TFSI)_0.2_(BF_4_)_0.8_. Similar to Emim(TFSI)_0.2_(BF_4_)_0.8_ as discussed earlier, cooling of Emim(TFSI)_0.8_(BF_4_)_0.2_ produces a glassy state structure.

**FIG. 6.**
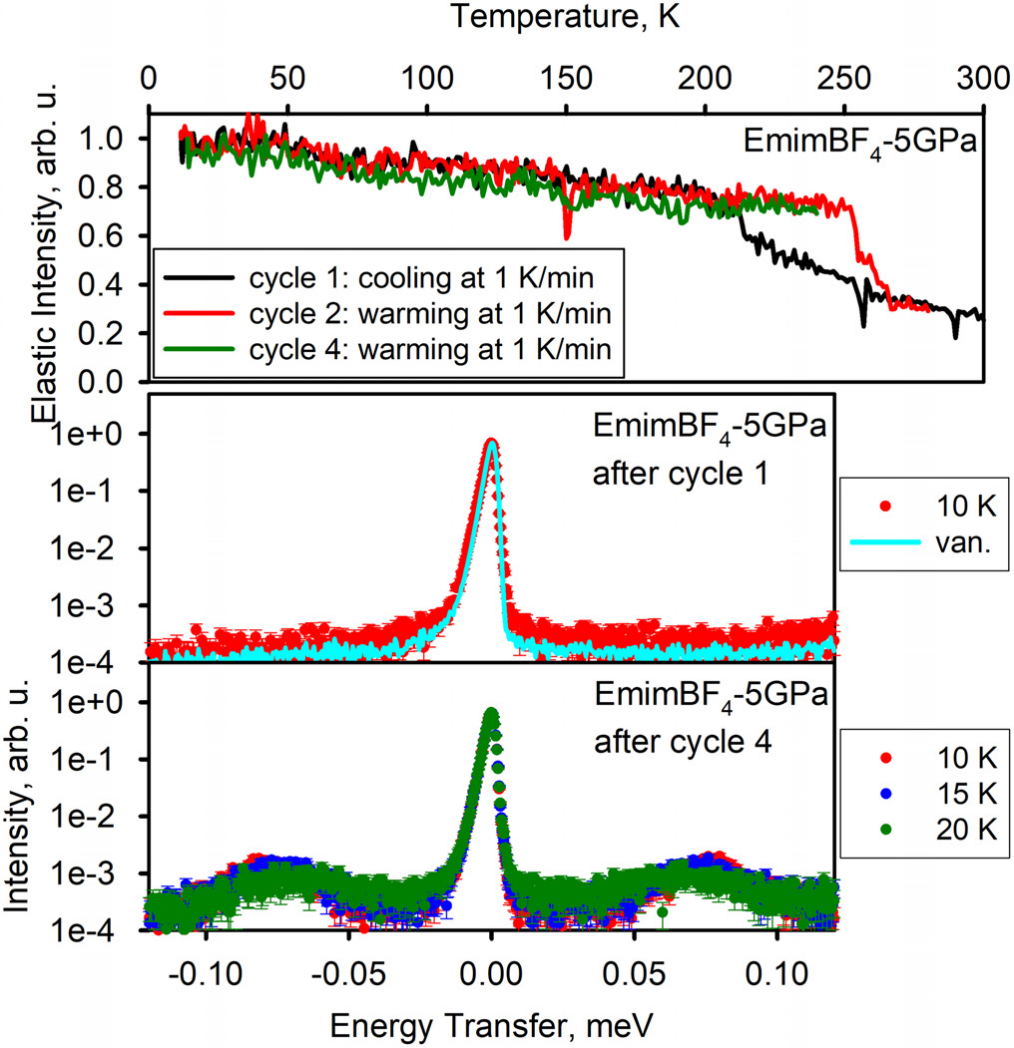
EmimBF_4_ pressure-cycled *ex situ* at 5 GPa. Top panel: elastic neutron scattering intensity as a function of temperature. Bottom panels: dynamics data—neutron scattering intensity as a function of neutron energy transfer measured at selected temperature points.

Figure 6 shows the results for EmimBF_4_ pressure-cycled *ex situ* at 5 GPa prior to the neutron scattering measurements. Initial cooling and subsequent warming (cycle 1 and cycle 2) show crystallization and melting steps at temperatures very different from the crystallization and melting temperatures observed for native EmimBF_4_, as one can see in Fig. 2. Furthermore, the measurement of the dynamics performed after the completion of cycle 1 shows the elastic line (resolution function, essentially), with no evidence of either well-defined tunneling peaks or quasi-elastic-type signal from tunneling in the glassy state. These observations suggest that pressure-cycled EmimBF_4_, even though visibly similar to the native EmimBF_4_, might have assumed a liquid structure different from the native liquid structure (e.g., due to ion dimerization, as was at first suggested for different pressure-cycled RTILs in quantum chemical calculations^33^ and then inferred from the experimental results^26^). Upon crystallization of this altered pressure-cycled liquid EmimBF_4_ into a different crystal structure, the tunneling peak positions are likely out of the dynamic range of the spectrometer. After completing cycle 2, the pressure-cycled sample was cooled down to 10 K (cycle 3) and warmed in a controlled manner up to ∼245 K (cycle 4). After cooling following the completion of cycle 4, the dynamics measurements of the sample exhibited methyl quantum tunneling peaks at ±75 *μ*eV, suggesting the restoration of the structure into which native EmimBF_4_ crystallizes (Fig. 2). That is, the pressure-induced metastable structure of pressure-cycled EmimBF_4_ must have been annealed into the standard crystal structure of the native EmimBF_4_.

## CONCLUSION

The samples of two common imidazolium-based room-temperature ionic liquids (RTILs), EmimBF_4_ and EmimTFSI, whose mixtures have been previously investigated for energy storage applications,^20,34^ were subjected to various treatments to make them either crystalline or amorphous at cryogenic temperatures. The 20:80 and 80:20 mixtures of EmimBF_4_ and EmimTFSI did not crystallize and instead formed a glass upon cooling from an ambient temperature at 1 K/min. Pure EmimBF_4_ crystallized under similar cooling conditions but could be quenched into a glass by inserting the ambient-temperature sample into the pre-cooled sample environment. On the contrary, pure EmimTFSI could not be turned into a glass under the same conditions. The EmimBF_4_ sample pressure-cycled at 5 GPa before the neutron scattering measurements crystallized into a structure different from the standard EmimBF_4_, although the crystal structure of the native EmimBF_4_ was restored by annealing. The samples that became glassy through either gradual cooling (the EmimBF_4_–EmimTFSI mixtures) or rapid quenching (pure EmimBF_4_) could be re-crystallized by annealing. Remarkably, there was a one-to-one correspondence between the state of the sample (crystalline vs amorphous) and the appearance of the methyl quantum tunneling peaks (well-defined vs distributed and giving rise to quasi-elastic-type continuous signal). We conclude that as long as the quantum tunneling energy of the methyl group falls into the dynamic range of the high energy-resolution neutron spectrometer, high energy-resolution inelastic neutron scattering can measure quantum tunneling of the methyl groups of the RTIL cations to probe the order-disorder (crystalline vs amorphous state). This approach utilizes standard protonated samples and, thus, is unhindered by the unavailability of deuterated analogs, which is the case for the overwhelming majority of RTILs. Given the high sensitivity of neutron scattering to H over heavier elements and the local character of rotational tunneling, this approach can potentially be applied to probing order-disorder in RTILs in confinement, where the application of diffraction-based methods may be challenging.

## AUTHORS' CONTRIBUTIONS

All authors contributed equally to this work.

## Data Availability

The data that support the findings of this study are available from the corresponding author upon reasonable request.
